# Supplementary material for: A Microarray, Validation, and Gene-Enrichment Approach for Assessing Differentially Expressed Circulating miRNAs in Obese and Lean Heart Failure Patients: A Case–Control Study
Source: Int J Mol Sci. 2025 Sep 27;26(19):9475. doi: 10.3390/ijms26199475 (PMC12524949; doi:10.3390/ijms26199475)
Supplement: Supplementary file 1 [file ijms-26-09475-s001.zip › S1_Table.pdf]

A Microarray, Validation, and Gene-Enrichment Approach for Assessing Differentially Expressed Circulating miRNAs in Obese and Lean Heart Failure Patients: A Case–Control Study

Douglas dos Santos Soares [1,2,†], Amanda Lopes [1,2,†], Mariana Recamonde-Mendoza [3,4], Rodrigo Haas Bueno [1,4], Raquel Calloni [5], Nadine Clausell [1,2], Santiago Alonso Tobar Leitão [1,2,*,‡] and Andreia Biolo [1,2,‡]

[1] Experimental and Molecular Cardiovascular Laboratory, Heart Failure and Cardiac Transplant Unit, Cardiology Division, Hospital de Clínicas de Porto Alegre, Porto Alegre 90035-903, Brazil
[2] Postgraduate Program in Cardiology and Cardiovascular Science, Federal University of Rio Grande do Sul, Porto Alegre 90040-060, Brazil
[3] Institute of Informatics, Universidade Federal do Rio Grande do Sul (UFRGS), Porto Alegre 90035-007, Brazil
[4] Bioinformatics Core, Hospital de Clínicas de Porto Alegre (HCPA), Porto Alegre 90035-003, Brazil
[5] Federal Institute of Education, Science and Technology of Rio Grande do Sul—Campus Gravataí, Gravataí 92412-240, Brazil
* Correspondence: tobar4@gmail.com
† These authors contributed equally to this work.
‡ These authors contributed equally to this work.

.

Supplemental material

Table S1 . miRNAs-genes-pathways signaling.

miRNAs	Genes	Pathways	Biological process
↑miR-451a, ↑miR-22-3p	AKT1	FoxO signaling pathway, Signaling pathways regulating pluripotency of stem cells, Prolactin signaling pathway, mTOR signaling pathway, Growth hormone synthesis, Secretion and action, Longevity regulating pathway, ErbB signaling pathway, B cell receptor signaling pathway.	Apoptosis, Carbohydrate metabolism, Glucose metabolism, Glycogen biosynthesis, Glycogen metabolism, Neurogenesis, Sugar transport, Translation regulation, Transport
↑miR-22-3p, ↑miR-548ac	GRB2	FoxO signaling pathway, Signaling pathways regulating pluripotency of stem cells, Prolactin signaling pathway, mTOR signaling pathway, Growth hormone synthesis, secretion and action, ErbB signaling pathway, B cell receptor signaling pathway.	Actin cytoskeleton reorganization, aging, anatomical structure formation involved in morphogenesis, branching involved in labyrinthine layer morphogenesis, cell differentiation, cellular response to ionizing radiation, epidermal growth factor receptor signaling pathway, insulin receptor signaling pathway, positive regulation of actin filament polymerization, positive regulation of actin filament polymerization, Ras protein signal transduction, receptor internalization, regulation of MAPK cascade, signal transduction in response to DNA damage.

↑miR-451a, ↑miR-22-3p	MAPK1	FoxO signaling pathway, Signaling pathways regulating pluripotency of stem cells, Prolactin signaling pathway, mTOR signaling pathway, Growth hormone synthesis, secretion and action, ErbB signaling pathway, B cell receptor signaling pathway.	Apoptotic process, B cell receptor signaling pathway, Bergmann glial cell differentiation, cardiac neural crest cell development involved in heart development, caveolin-mediated endocytosis, cell cycle, cell surface receptor signaling pathway, cellular response to amino acid starvation, cellular response to cadmium ion, cellular response to DNA damage stimulus, cellular response to dopamine, cellular response to granulocyte macrophage colony-stimulating factor stimulus, cellular response to reactive oxygen species, cellular response to tumor necrosis factor, chemical synaptic transmission, chemotaxis, cytosine metabolic process, ERBB signaling pathway, ERK1 and ERK2 cascade, face development, intracellular signal transduction, labyrinthine layer blood vessel development, learning or memory, lipopolysaccharide-mediated signaling pathway, long-term synaptic potentiation, lung morphogenesis, mammary gland epithelial cell proliferation, negative regulation of cell differentiation, outer ear morphogenesis, peptidyl-serine phosphorylation, peptidyl-threonine phosphorylation, positive regulation of gene expression, positive regulation of macrophage chemotaxis, positive regulation of macrophage proliferation, positive regulation of peptidyl-threonine phosphorylation, positive regulation of telomerase activity, positive regulation of telomere capping, positive regulation of telomere maintenance via telomerase, protein phosphorylation, regulation of cellular pH, regulation of cytoskeleton organization, regulation of early endosome to late endosome transport, regulation of Golgi inheritance, regulation of ossification, regulation of protein stability, regulation of stress-activated MAPK cascade, response to epidermal growth factor, response to exogenous dsRNA, response to nicotine, signal transduction, stress-activated MAPK cascade, T cell receptor signaling pathway, thymus development, thyroid gland development, trachea formation.

↑miR-22-3p, ↑miR-548ac	IGF1R	FoxO signaling pathway, Signaling pathways regulating pluripotency of stem cells, mTOR signaling pathway, Longevity regulating pathway.	Cellular response to transforming growth factor beta stimulus, cellular senescence, cerebellum development, dendritic spine maintenance, establishment of cell polarity, estrous cycle, glucose homeostasis, hippocampus development, immune response, insulin receptor signaling pathway, insulin-like growth factor receptor signaling pathway, negative regulation of apoptotic process, negative regulation of cholangiocyte apoptotic process, negative regulation of hepatocyte apoptotic process, negative regulation of MAPK cascade, negative regulation of muscle cell apoptotic process, peptidyl-tyrosine autophosphorylation, phosphatidylinositol 3-kinase signaling, phosphatidylinositol-mediated signaling, positive regulation of axon regeneration, positive regulation of cell migration, positive regulation of cell population proliferation, positive regulation of cold-induced thermogenesis, positive regulation of cytokinesis, positive regulation of DNA metabolic process, positive regulation of kinase activity, positive regulation of MAPK cascade, positive regulation of osteoblast proliferation, positive regulation of phosphatidylinositol 3-kinase signaling, positive regulation of protein kinase B signaling, positive regulation of protein-containing complex disassembly, positive regulation of smooth muscle cell proliferation, positive regulation of steroid hormone biosynthetic process, protein autophosphorylation, regulation of JNK cascade, response to ethanol, response to L-glutamate, response to nicotine, response to vitamin E, signal transduction, transcytosis, transmembrane receptor protein tyrosine kinase signaling pathway.

↑miR-22-3p, ↑miR-548ac	PTEN	FoxO signaling pathway, mTOR signaling pathway.	adult behavior, angiogenesis, apoptotic process, brain morphogenesis, canonical Wnt signaling pathway, cardiac muscle tissue development, cell migration, cell motility, cellular response to electrical stimulus, cellular response to hypoxia, central nervous system development, central nervous system myelin maintenance, central nervous system neuron axonogenesis, dendritic spine morphogenesis, dentate gyrus development, dephosphorylation, endothelial cell migration, forebrain morphogenesis, heart development, inositol phosphate dephosphorylation, learning or memory, locomotor rhythm, locomotory behavior, long-term synaptic potentiation, male mating behavior, maternal behavior, multicellular organismal response to stress, negative regulation of apoptotic process, negative regulation of axon regeneration, negative regulation of axonogenesis, negative regulation of cardiac muscle cell proliferation, negative regulation of cell aging, negative regulation of cell cycle G1/S phase transition, negative regulation of cell migration, negative regulation of cell population proliferation, negative regulation of cell size, negative regulation of cyclin-dependent protein serine/threonine kinase activity, negative regulation of dendritic spine morphogenesis, negative regulation of epithelial cell proliferation, negative regulation of epithelial to mesenchymal transition, negative regulation of ERK1 and ERK2 cascade, negative regulation of excitatory postsynaptic potential, negative regulation of focal adhesion assembly, negative regulation of G1/S transition of mitotic cell cycle, negative regulation of keratinocyte migration, negative regulation of myelination, negative regulation of neuron projection development, negative regulation of organ growth, negative regulation of peptidyl-serine phosphorylation, negative regulation of phosphatidylinositol 3-kinase signaling, negative regulation of protein kinase B signaling, negative regulation of protein phosphorylation, negative regulation of ribosome biogenesis, negative regulation of synaptic vesicle clustering, negative regulation of vascular associated smooth muscle cell proliferation negative regulation of wound healing, spreading of epidermal cells, negative regulation of wound healing, spreading of epidermal cells, neuron-neuron synaptic transmission, phosphatidylinositol 3-kinase signaling, phosphatidylinositol biosynthetic process, phosphatidylinositol dephosphorylation, positive regulation of cell population proliferation, positive regulation of DNA-binding transcription factor activity, positive regulation of ERK1 and ERK2 cascade, positive regulation of excitatory postsynaptic potential, positive regulation of TRAIL-activated apoptotic signaling pathway, positive regulation of ubiquitin protein ligase activity, positive regulation of ubiquitin-dependent protein catabolic process, postsynaptic density assembly, prepulse inhibition, presynaptic membrane assembly, prostate gland growth, protein dephosphorylation, protein kinase B signaling, protein stabilization, regulation of B cell apoptotic process, regulation of cellular component size, regulation of myeloid cell apoptotic process, regulation of neuron projection development, regulation of protein kinase B signaling, regulation of protein stability, regulation of synaptic transmission, GABAergic, rhythmic synaptic transmission, social behavior, synapse assembly, synapse maturation.

↑miR-22-3p, ↑miR-548ac	ESR1	Prolactin signaling pathway.	Transcription, Transcription regulation
↑miR-22-3p, ↑miR-548ac	HSPA1B	Longevity regulating pathway.	ATP metabolic process, cellular heat acclimation, cellular response to heat, cellular response to oxidative stress, cellular response to steroid hormone stimulus, cellular response to unfolded protein, chaperone cofactor-dependent protein refolding, mRNA catabolic process, negative regulation of apoptotic process, negative regulation of cell death, negative regulation of cell growth, negative regulation of cell population proliferation, negative regulation of extrinsic apoptotic signaling pathway in absence of ligand, negative regulation of inclusion body assembly, negative regulation of protein ubiquitination, positive regulation of erythrocyte differentiation, positive regulation of gene expression, positive regulation of interleukin-8 production, positive regulation of microtubule nucleation, positive regulation of NF-kappa B transcription factor activity, positive regulation of nucleotide-binding oligomerization domain containing 2 signaling pathway, positive regulation of proteasomal ubiquitin-dependent protein catabolic process, positive regulation of tumor necrosis factor-mediated signaling pathway, protein refolding, protein stabilization, regulation of mitotic spindle assembly, regulation of protein ubiquitination, vesicle-mediated transport.
↑miR-451, ↑miR-548ac	MAP3K1	Growth hormone synthesis, secretion and action.	Cellular response to mechanical stimulus, Fc-epsilon receptor signaling pathway, protein phosphorylation.
↑miR-22-3p, ↑miR-548ac	ZFHX3	Signaling pathways regulating pluripotency of stem cells.	Brain development, circadian regulation of gene expression, muscle organ development, negative regulation of myoblast differentiation, negative regulation of transcription by RNA polymerase II, positive regulation of cell adhesion, positive regulation of myoblast differentiation, positive regulation of transcription by RNA polymerase II, positive regulation of transcription, DNA-templated, regulation of cell cycle, regulation of locomotor rhythm, regulation of neuron differentiation, regulation of transcription by RNA polymerase II, regulation of transcription, DNA-templated, response to transforming growth factor beta.
